# P-1522. Optimal Dosing and Outcomes In Patients Treated for *Acinetobacter Baumannii* Pneumonia and Bacteremia

**DOI:** 10.1093/ofid/ofae631.1691

**Published:** 2025-01-29

**Authors:** Pramodini Kale-Pradhan, Sarah Channey, Sabrina Adragna, Christopher Giuliano, Merideth Coyle, Leonard B Johnson

**Affiliations:** Wayne State University Eugene Applebaum College of Pharmacy and Health Sciences, Detroit, Michigan; Wayne State Unvieristy Eugene Applebaum College of Pharmacy and Health Sciences, Detroit, Michigan; Wayne State University Eugene Applebaum College of Pharmacy and Health Sciences, Detroit, Michigan; Wayne State University Eugene Applebaum College of Pharmacy and Health Sciences, Detroit, Michigan; Cook County Health, Chicago, Michigan; Ascension St. John Hospital, Detroit, Michigan

## Abstract

**Background:**

*Acinetobacter baumannii*

(ABM) is a gram-negative coccobacillus associated with infections in healthcare facilities and has a high mortality rate. Guidelines recommend nine grams ampicillin/sulbactam (amp/sulb) every eight hours for moderate to severe ABM infections. We assessed the dosing and effectiveness of amp/sulb in the treatment of ABM pneumonia and bacteremia.

**Methods:**

We conducted a retrospective cohort study of patients receiving high dose (≥ 3.1 gms q4h or CrCl < 30mL/min) compared to low dose (< 3.1gms q6h or if CrCl ≥ 30 mL/min) amp/sulb for ABM bacteremia and pneumonia infections from 2017-2022. Patients were also included in the high dose group if CrCl < 30 ml/min and were receiving 3.1 gms q4h. The primary outcome was all cause mortality during hospitalization. The secondary outcome was ventilator free days.

**Results:**

Of the 277 patients screened; 173 did not receive amp/sulb, 38 did not have ABM infection, 10 had penicillin allergy and 4 were duplicate patients. Of the 52 included patients, 18 (34.6%) received low dose and 34 (65.3%) received high dose. The mean age of the patients was 64.8 and 71.1% were males. The most common infection was pneumonia 27(51.9%) followed by 25 (48.1%) treated for bacteremia. Of the low dose group three patients (16.6%) died compared to four patients (11.7%) in the high dose group during their admission. Four patients (22.2%) and five patients (14.7%) in low dose and high dose groups were discharged to hospice respectively. Eleven of the 18 patients (61.1%) and 25 of 34 (73.6%) patients were discharged to home following resolution of ABM infection in the low and high dose groups respectively.

**Conclusion:**

No difference was found between the low and high dose amp/sulb for the treatment of ABM pneumonia and bacteremia in patient outcomes. Further studies are needed to confirm these findings.

**Disclosures:**

**All Authors**: No reported disclosures

